# Dependency-based long short term memory network for drug-drug interaction extraction

**DOI:** 10.1186/s12859-017-1962-8

**Published:** 2017-12-28

**Authors:** Wei Wang, Xi Yang, Canqun Yang, Xiaowei Guo, Xiang Zhang, Chengkun Wu

**Affiliations:** School of Computer Science, National University of Defense Technology, Changsha, 410073 China

**Keywords:** Relation extraction, Long short term memory, Dependency tree, Data imbalance

## Abstract

**Background:**

Drug-drug interaction extraction (DDI) needs assistance from automated methods to address the explosively increasing biomedical texts. In recent years, deep neural network based models have been developed to address such needs and they have made significant progress in relation identification.

**Methods:**

We propose a dependency-based deep neural network model for DDI extraction. By introducing the dependency-based technique to a bi-directional long short term memory network (Bi-LSTM), we build three channels, namely, Linear channel, DFS channel and BFS channel. All of these channels are constructed with three network layers, including embedding layer, LSTM layer and max pooling layer from bottom up. In the embedding layer, we extract two types of features, one is distance-based feature and another is dependency-based feature. In the LSTM layer, a Bi-LSTM is instituted in each channel to better capture relation information. Then max pooling is used to get optimal features from the entire encoding sequential data. At last, we concatenate the outputs of all channels and then link it to the softmax layer for relation identification.

**Results:**

To the best of our knowledge, our model achieves new state-of-the-art performance with the F-score of 72.0% on the DDIExtraction 2013 corpus. Moreover, our approach obtains much higher Recall value compared to the existing methods.

**Conclusions:**

The dependency-based Bi-LSTM model can learn effective relation information with less feature engineering in the task of DDI extraction. Besides, the experimental results show that our model excels at balancing the Precision and Recall values.

## Background

Drug-drug interaction is a situation in which one drug influences the level or activity of another drug when both are taken in combination. Such interactions may result in either synergistic or antagonistic effect. A specific instance of antagonistic effect is adverse drug reaction (ADR), which has been a growing problem in hospital medicine. Those unexpected side effects caused by ADR are serious health hazards and sometimes even result in death. A slew of studies have pointed to the recent swift growth of the numbers of ADRs [1]. It is reported that more than 300,000 deaths are caused by ADRs per year in the USA and Europe [2, 3]. More seriously, according to data from Centers for Disease Control and Prevention, adverse drug reactions harm anywhere from 1.9 to 5 million inpatients per year. Owing to the aging of population and the rise in more people taking multiple medications, the problem likely continues to get worse. As a result, the detection of DDIs have been taken seriously by pharmaceutical companies and drug agencies in drug safety and healthcare management.

So far, there are multiple databases supporting the healthcare professionals in recognizing adverse effects of drugs, such as DrugBank [4], Stockley [5]. However, the time and labor-consuming to manually keep updating them with the rapidly growing volume of biomedical literatures are unacceptable, which means massive amount of valuable DDIs remain hidden in the unstructured published articles, scientific journals, books and technical reports [1]. Therefore, there is a sharp increase in interest in automatic extraction of DDIs information from biomedical texts.

Realizing the importance of interaction information between two drugs, DDI extraction has been developed as a widely studied relation extraction task in natural language processing [6]. Various methods have been proposed aiming at DDI extraction. Existing approaches can be roughly classified into pattern-based methods and machine learning-based methods [7]. Pattern-based methods use manually defined patterns to identify DDIs, whereas machine learning-based [8–10] methods learn effective features over the annotated corpora for relation extraction. Early studies in DDI extraction are almost all pattern-based. For examples, IS Bedmar obtained the patterns with the help of a pharmacist [11], Blasco et al. extracted the patterns by Maximal Frequent Sequences [12] and Segura-Bedmar et al. defined a set of domain-specific rules for DDI extraction.

In general, machine learning-based methods have shown better performance and better portability than pattern-based methods and can be easily extended to new dataset, even new domain [13]. However, machine learning-based methods are limited on the annotated corpora, which usually take much time and labor to accomplish the annotation. In recent years, based on a benchmark corpus, the DDI corpus shared by DDIExtraction challenges in 2011 and 2013 [14, 15], various machine learning-based approaches have been proposed to accomplish the task of DDI extraction. DDIExtraction 2011 challenge focused on the detection of DDIs, while DDIExtraction 2013 challenge required DDIs being classified into four predefined DDI types: Advice, Effect, Mechanism and Int. Roughly, existing methods of DDI extraction can be divided into two categories: one-stage and two-stage methods. In one-stage methods [6, 16–19], a multiclass classifier is built to directly classify each candidate DDI instance into one of the five types, including Advice, Effect, Mechanism, Int and Negative class. As the name suggests, the two-stage methods [20–22] split the problem into two stages: first, a binary classifier is built to recognize all candidate instances into positive instances or negative instances, then only the positive instances are considered to be classified into one of the four predefined DDI types. A further comparison among these methods reveals that deep neural network models, including Convolutional Neural Network (CNN) [23, 24], and sequential neural networks such as Recurrent Neural Network (RNN) [25] and Long Short Term Memory Network (LSTM) [26, 27], perform better than models based on Support Vector Machine (SVM) with linear or non-linear kernel in relation classification. Effective relation features can be learned by these powerful deep neural network models without complicated feature engineering [19].

Although various approach have been proposed, the research about DDI extraction is still in its infancy and there is still much room for improvement on its performance [22]. In this work, we aim to construct a relation extraction model for drug-drug interaction by integrating deep neural network and less but more effective features. A key feature of our work is that we apply the dependency-based technique to a deep neural network, bi-directional LSTM network, which has shown significant power in processing long sequential data. We realize three separate channels equipped with Bi-LSTM, named as Linear channel, DFS channel and BFS channel, in our model to learn valuable information for DDI extraction. Here Linear channel utilizes a Bi-LSTM for encoding linear sequence, while DFS channel and BFS channel use the Bi-LSTMs to encode the corresponding dependency-based sequential data. All of these three channels are constructed with three network layers from bottom up, including embedding feature layer, LSTM layer and max pooling layer. In the embedding feature layer, distanced-based features are linked to the linear channel, and dependency-based features are linked to the DFS channel and the BFS channel. Both of these two kinds of features are initialized with syntax word embedding or random word embedding. We make a detailed and exhaustive comparative study of such two kinds of word embedding methods in the discussion part. After that, in the LSTM layer, a Bi-LSTM is instituted in each channel to better capture relation information. Instead of concatenating the outputs of forward LSTM layer and backward LSTM layer, we define a new and simple rule to combine the outputs obtained by encoding the sequence in different direction. Then we employ the max pooling method to get optimal features from the entire encoding sequential data in the max pooling layer. Lastly, the outputs of all channels are concatenated together and then fed to the softmax layer for relation classification.

To the best of our knowledge, our model achieves new state-of-the-art performance with the F-score of 72.0%. Moreover, our approach obtains much higher Recall value compared to the existing methods. Namely, our model excels at balancing the Precision and Recall values, leading to a higher F-score.

## Methods

**Fig. 1:**
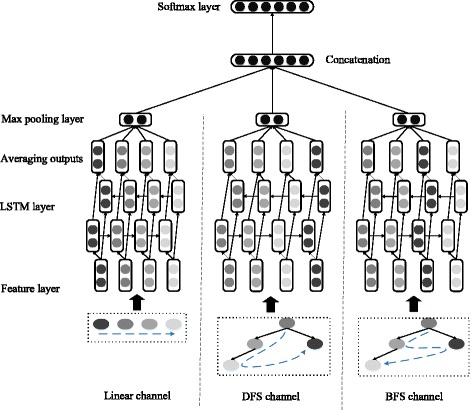
The framework of our model

We propose a LTSM based multi-classification model aiming at the task of DDI extraction. All pairs of drugs in each sentence are either recognized as non-interacting pair, or classified into one of the predefined types of DDIs. The framework of our model is shown in Fig. 1. The first layer constructs two types of embedding features as input for LSTM layer, including distance-based feature and dependency-based feature. Each type of features is linked to the corresponding channel in LSTM layer, then the encoding outputs from different channels are concatenated to extract the relations. The components of our model are described in detail in the following parts.

### Embedding feature layer

In our model, we extend two kinds of discrete features, including distance-based features and dependency-based features, to represent each word in the sentence.

#### Distance-based feature

we follow the previous studies [24] to characterize a word with the position features consisting of two relative distances. Thus, each word in a sentence is represented with[*w*, *D*_1_, *D*_2_], where *w* is the exact word, *D*_1_ and *D*_2_ are relative distances from current word to the first drug and the other drug, respectively. This way the value of either *D*_1_ or *D*_2_ would be zero for the corresponding drug names. Take the following instance in which the pair of drugs are highlighted in italic as an example.

“The findings suggest that the dosage of *S-ketamine* should be reduced in patients receiving *ticlopidine*”. The relative distances of the word “suggest” to the pair of drugs are 5 and 12, respectively. In terms of the drug name “*S-ketamine*”, the distance values would be 0 and 7.

#### Dependency-based feature

**Fig. 2:**
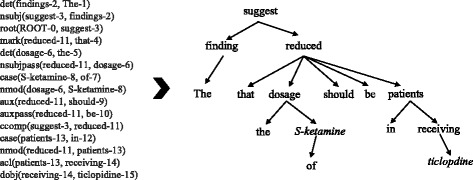
An example of the typed dependency representation and the corresponding dependency tree

A dependency relationship is an asymmetric binary relation between two words in a sentence [28]. Normally with the dependency relationships, all words in a sentence are connected, called the dependency structure of the sentence. In this way, a sentence is converted into a dependency tree. We utilize Stanford Parser [29] to get the dependency relation between words in a sentence. For example, consider the text: The findings suggest that the dosage of *S-ketamine* should be reduced in patients receiving *ticlopidine*. The typed dependency representation and the corresponding dependency tree are given as shown in Fig. 2. Take “nsubj(suggest-3, findings-2)” as an example, node “suggest” is the governor of node “findings” and “nsubj” represents the grammatical relation between them.

In Fig. 2, the root (the word “suggest”) of the dependency tree plays a decisive role in recognizing the relation between two drugs *(S-ketamine* and *ticlopdine*). It is consistent with the intuition that more attention should be paid to the words surrounding the root in the tree, assuming that the closer words contain more information for the relation extraction. Hence, similar to distance-based feature, we construct the dependency-based feature by representing each word with [*w*, *L* − *L*_1_, *L* − *L*_2_], where *w* is the exact word, *L* is the shortest distance from current node to the root in the dependency tree. *L* − *L*_1_ and *L* − *L*_2_ represent the differences between the distance values in terms of current node and the targeted drugs.

Syntax word embedding based on word2vec [30] and random word embedding are respectively employed in mapping the words to real-valued vectors. Besides, the distance values are mapped to a ten bit binary vector. Then the embedding distance-based feature and dependency-based feature constitute the first layer of our model, separately being linked to the corresponding channel in LSTM layer.

### LSTM layer

LSTM is an outstanding model for modeling long sequential data. In this layer, we build three separate channels in this paper to further process the corresponding type of embedding features of a sentence into specific sequential data. These three channels are defined as follows:

- **Linear channel**: in this channel we generate the sequential data with distance-based features in original order.
- **DFS channel**: based on the dependency tree, we generate the sequential data with dependency-based features by going through the tree using depth first search.
- **BFS channel**: similar to DFS channel but traversing the tree using breadth first search, the sequential data is produced with dependency-based features.

**Fig. 3:**
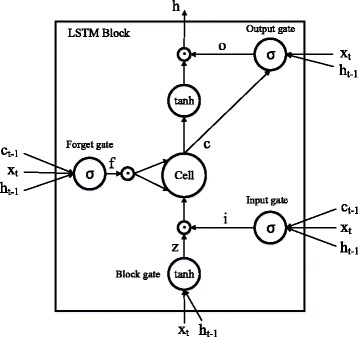
LSTM memory block

Each of these three channels is equipped with a bi-directional LSTM model to process the corresponding sequential data. The bi-directional LSTM model contain two parallel LSTM layers, including forward LSTM layer and backward LSTM layer. Basing on recurrent neural network architecture, LSTM model aims at overcoming the long-term dependencies problem. More precisely, LSTM model introduces a new structure of the memory block with a memory cell (*c*_*t*_) and three multiplicative gates, including the input gate (*i*_*t*_), output gate (*o*_*t*_), and forget gate (*f*_*t*_), to deal with the difficulty lying in the vanishing gradient problem which means the back propagated error either blows up or decays exponentially. Respectively, the activation of the input gate multiplies the input to the cells, the output gate multiplies the output to the net, and the forget gate multiplies the previous cell values. The illustration of a LSTM memory block is shown in Fig. 3. Let ${x}_1^{ch},{x}_2^{ch},\dots, {x}_i^{ch},\dots, {x}_m^{ch}$ be the sequential data, where ${x}_i^{ch}$ represents a feature vector of the word, *m* is the length of sentence and *ch* represents the corresponding channel. Let ${h}_t^f$ and ${c}_t^f$ be current hidden vector and cell vector respectively in forward LSTM layer. Similarly, current hidden vector and cell vector in backward LSTM layer are respectively denoted as ${h}_t^b$ and ${c}_t^b$. At each time step, ${h}_t^f$ and ${c}_t^f$ is computed based on the ${h}_{t-1}^f$ and ${c}_{t-1}^f$ of LSTM block. The detail operation is defined as follows:

$$ {\displaystyle \begin{array}{c}{i}_t=\sigma \left({W}_{xi}{x}_t+{W}_{hi}{h}_{t-1}+{W}_{ci}{c}_{t-1}+{b}_i\right)\\ {}{f}_t=\sigma \left({W}_{xf}{x}_t+{W}_{hf}{h}_{t-1}+{W}_{cf}{c}_{t-1}+{b}_f\right)\\ {}{z}_t=\tanh \left({W}_{xc}{x}_t+{W}_{hc}{h}_{t-1}+{b}_c\right)\\ {}{c}_t={f}_t\cdot {c}_{t-1}+{i}_t\cdot {z}_t\\ {}{o}_t=\sigma \left({W}_{xo}{x}_t+{W}_{ho}{h}_{t-1}+{W}_{co}{c}_t+{b}_o\right)\\ {}{h}_t={o}_t\cdot \tanh \left({c}_t\right)\end{array}} $$

Where *σ* is sigmoid activation function, *b* is the bias term, · is element-wise multiplication and *W*_(.)_ are learning parameters of LSTM model. Accordingly, ${h}_t^b$ can be computed by reversing the sequential data.

Instead of concatenating ${h}_t^f$ and ${h}_t^b$ to represent word’s encoding information (*z*_*t*_) in most of previous studies, we average ${h}_t^f$ and ${h}_t^b$ as follow:

$$ {z}_t=\left({h}_t^f+{h}_t^b\right)/2 $$

### Max pooling layer

The scope of pooling layer is to obtain a fixed length vector by performing feature selection. We choose max pooling to get the maximum over the entire sequence. Let *z*_1_, *z*_2_, …, *z*_*t*_, …, *z*_*m*_ be the sequence of the output of the corresponding channel in LSTM layer and $<{v}_t^1,{v}_t^2,\dots, {v}_t^d>$ be the vector of *z*_*t*_. The result of max pooling would be:

$$ z=<\max \left({v}^1\right),\max \left({v}^2\right),\dots, \max \left({v}^d\right)> $$

Where max(.) is the function of taking the maximum value of each dimension wise and *d* is the dimension. Then we concatenate all channels’ outputs after max pooling is done respectively.

$$ Z={z}^{linear}\oplus {z}^{DFS}\oplus {z}^{BFS} $$

### Softmax layer

We non-linearize the output of pooling layer by using tanh activation. After that we set a softmax layer with dropout layer, which makes the model more robust by avoiding overfitting. The detail operation is defined as follows:

$$ {\displaystyle \begin{array}{c}{h}^s=\tanh \left({h}^p\right)\\ {}p\left(y|x\right)= Soft\max \left({W}^s{h}^s+{b}^s\right)\end{array}} $$

Where *h* ^*p*^ is the output of max pooling layer, *W* is the softmax matrix and *b* is the bias parameter.

### Model training

The parameters including weights and biases of the entire network are updated by backpropagation through time. We use the cross entropy loss function and Adam optimization [31] with gradient clipping, parameter averaging and L2-regularization while training our model. In terms of the imbalanced class distribution problem, we employ two enhancements, negative instance filtering and training set sampling, which are described in detail in the following section.

### Dataset description

Our Model is evaluated on a benchmark corpus, the DDI corpus [1], which is shared by the 2013 DDIExtraction challenge. The DDI corpus is a valuable gold-standard for those researches focusing on the analysis of pharmacological substances, specifically for those working on DDI relation extraction. This dataset consists of 1017 texts, including 784 texts selected from the DrugBank database and 233 abstracts on the subject of DDI selected from the MEDLINE database. The corpus is split into training and test instances provided by sentences. All pairs of drugs in each sentence are manually annotated with the following four kinds of DDI types:

- **Advice**: this type is assigned when a recommendation or advice related to the concomitant use of two drugs is given, e.g., “If at all possible guanethidine should be discontinued well before minoxidil is begun”.
- **Effect**: this type is assigned when the effect of a DDI between two drugs is described. For example, “Decreased seizure threshold has been reported in patients receiving CYLERT concomitantly with antiepileptic medications”.
- **Mechanism**: this type is assigned when the sentence describes a pharmacokinetic mechanism. For example, “Oral hypoglycemic agents Oxandrolone may inhibit the metabolism of oral hypoglycemic agents”.
- **Int**: this type is assigned when a DDI is simply stated in the sentence without providing any other information, e.g., “Interactions for Vitamin B1 (Thiamine): Loop Diuretics”.

Before feeding the dataset to our model, a series of preprocessing operations are done: drug blinding, negative instance filtering and training set sampling.

### Drug blinding on dataset

For keeping the generalization of our model, the two drugs in pair are respectively replaced with “DRUG_1” and “DRUG_2” in turn by following the earlier studies [6, 22], and all the other drugs in the same sentence are replaced by “DRUG_N”. For instance, the DDI candidates in the sentence “The CNS-depressant effect of *propoxyphene* is additive with that of other *CNS depressants*, including *alcohol*” are blinded as shown in Table 1.

**Table 1:** An example of drug blinding

Drug candidate	Sentence with drug blinding
(*propoxyphene, CNS depressant*)	The CNS-depressant effect of *DRUG_1* is additive with that of other *DRUG_2*, including *DRUG_N*
(*propoxyphene, alcohol*)	The CNS-depressant effect of *DRUG_1* is additive with that of other *DRUG_N*, including *DRUG_2*
(*CNS depressant, alcohol*)	The CNS-depressant effect of *DRUG_N* is additive with that of other *DRUG_1*, including *DRUG_2*

After drug blinding, all words are converted to lowercase and sentences are tokenized using the Natural Language Toolkit [32].

### Dataset balancing

Having 1:5.8 ratio for training set and 1:4.8 ratio for test set on positive instances and negative instances, the DDI corpus suffers from the imbalanced class distribution problem, which will significantly affect the performance of the classification model. To alleviate it, we first filter out the negative instances on the entire dataset based on the predesigned rules. Then concerned on the training data, sampling is expected to correct the imbalanced issue.

#### Negative instance filtering

The previous studies [22, 33] has shown that negative instance filtering makes sense on constructing a less imbalanced corpus and has positive impact on classification model. Therefore, we define the following rules to remove the possible negative instances:

- **Rule 1**: the two targeted drugs share the same name. In such case, exact string matching is made use of to filter out the corresponding instances.
- **Rule 2**: one drug is a special case of the other drug. To satisfy this criteria, we apply the patterns (e.g., “DRUG_1 (DRUG_N* DRUG_2)”, “DRUG_1 such as DRUG_N* DRUG_2”) using regular expression to remove such case. An example in which the pair of drugs are highlighted in italic is given as follow: “A variety of *antiarrhythmics* such as *quinidine* or propranolol were also added, sometimes with improved control of ventricular ectopy.”
- **Rule 3**: the two candidate drugs appear in the same coordinate structure. Again, several patterns, such as “DRUG_1 DRUG_N* and*|or* DRUG_2”, are used to remove such instances. For example, the following instance will be filtered out according to rule 3: “Sulfamethizole may increase the effects of *barbiturates*, *tolbutamide*, and uricosurics.”

#### Training set sampling

Generally, sampling is expected to correct the imbalance of the dataset since the majority class is more dominant than the minority class in satisfying the objective function of the machine learning model [34]. There are two effective methods to adjust the class distribution of the dataset: under sampling and oversampling. The former one decreases majority cases, while the latter one increases minority cases.

As shown in Table 2, after negative instance filtering, having 94.0:1 ratio on Negative and Int instances, the training set of the DDI corpus still exists a serious imbalanced issue. Hence, we employing under sampling and oversampling in Negative and Int instances, respectively, to obtain a more balanced training set. Let ${X}_{neg}^f$ and ${X}_{\mathrm{int}}^f$ be the outputs of Negative instances and Int instances in training set after negative instances filtering, then the outputs of sampling would be:

$$ {\displaystyle \begin{array}{c}{X}_{neg}^s= Sfun\left(\alpha, {X}_{neg}^f\right)\\ {}{X}_{\mathrm{int}}^s=\sum \limits_{k=1}^K Sfun\left(\beta, {X}_{\mathrm{int}}^f\right)\end{array}} $$

**Table 2:** The statistics of the DDI corpus

	Training set	Training set filtering	Test set	Test set filtering
Negative	23,371	17,297	4737	3335
Advice	1319	1315	302	301
Effect	1687	1677	360	357
Mechanism	826	821	221	221
Int	189	184	96	96
Total	27,792	21,294	5716	4310
Ra.	1:5.8	1:4.3	1:4.8	1:3.4

Note The Ra. denotes the ratio between positive instances and negative instances

Where *α*, *β* are sampling ratios, *Sfun*(.) is the function of sampling based on sampling ratio and *K* is sampling times. As under sampling might discard valuable samples, it is done within every interaction to obtain different sampling outputs while training our model. In this way, we expect to cover all the negative cases. Meanwhile, to overcome the overfitting of the corresponding cases caused by oversampling, the ratio of dropout, is set up in our model to eliminate the outputs of LSTM cells randomly.

## Results and discussion

### Experimental settings

Our model is coded with Python language using Tensorflow [35] package and is evaluated with the same scheme as used in the DDIExtraction 2013 chanllenge [15], including Precision (P), Recall (R) and F-score (F). As our model adopts the manner of one-stage, all candidate DDI instances are classified into five types, including Advice, Effect, Mechanism, Int and Negative class.

We use two different methods to initialize the word embedding matrix: syntax word embedding based on word2vec and random word embedding method. The syntax word embedding used in our experiments is pre-trained by the Skip-gram algorithm [36] on about 14-gigabyte unannotated article titles and abstracts extracted from MEDLINE [37] database. Following the previous studies [38], we look up the syntax word embedding matrix to get the word embedding of known words that present in the vocabulary, and randomly initialize the word embedding of unknown words that do not present in the vocabulary. We call the model using syntax word embedding with the name of DLSTM^1^. On the other hand, in the random word embedding method, denoted as DLSTM^2^, we initialize the word embedding of all words with random real values from −1 to 1.

In this work, we propose a relation classification model based on bi-directional long short term memory network. The hyper parameters used in our model are summarized in Table 3.

**Table 3:** The hyper parameters of our model

Parameter	Description	Value
*dw*	Dimension of word embedding	100
*dp*	Dimension of distance embedding	10
*num*	The number of hidden units	300
*ρ*	The ratio of dropout	0.7
*l*_2_	The L2 regularization	0.001
*l*_*a*_	The learning rate of Adam optimizer	0.01
*α*	The ratio of under sampling	0.5
*β*	The ratio of oversampling	0.5
*K*	The times of oversampling	6

We use the recent methods as baselines, which include linear methods (Kim, UTurku), kernel methods (FBK-irst, NIL_UCM) and neural network methods (CNN, SCNN^1^, SCNN^2^, CNN&DCNN, B-LSTM, AB-LSTM and Joint AB-LSTM). Briefly descriptions about these methods are given as follows:

- **Kim** [33] built a linear SVM classifier relying on a rich set of lexical and syntactic features.
- **UTurku** [21] used the features extracted from dependency parsing and domain dependent resources to realize the Turku event extraction system for DDI extraction.
- **FBK-irst** [39] was a two-stage method of relation extraction. A hybrid kernel was used in the model to train a classifier with syntax tree and dependency tree features.
- **NIL_UCM** [40] used a multiclass SVM as kernel methods relying on lexical, morphosyntactic and parse tree features.
- **CNN** [6] employed the convolutional neural network in DDI extraction without manually defined features.
- **SCNN** ^**1**^ and **SCNN** ^**2**^ [22] utilized features based on PoS tags and dependency tree to train the convolution neural network with max pooling layer.
- **CNN&DCNN** [41] designed a simple rule to combine convolutional neural network and dependency-based convolutional neural network.
- **B-LSTM, AB-LSTM and Joint AB-LSTM** [42] utilized word and distance embedding as latent features with no feature engineering and learnt higher level features representation through bidirectional long short term memory network.

### Comparison with baseline methods

The performance among our models and baseline methods is shown in Table 4. As can be seen from it, the neural network methods outperform the linear methods and the kernel methods in Precision, Recall and F-score. It is indicated that deep neural networks show more significant power in relation extraction with less or no handcrafted features. To the best of our knowledge, DLSTM^1^ model achieves new state-of-the-art performance with the F-score of 72.0%. There is 5% of relative improvement on F-score when comparing with the best result (67% in Kim method) of linear methods and kernel methods. Furthermore, the models, including DLSTM^1^, DLSTM^2^, B-LSTM, AB-LSTM and Joint AB-LSTM, that are equipped with long short term memory network perform better than those models that are equipped with convolutional neural network, which is consistent with the intuition that long short term memory network outperforms in processing long sequential data due to its nature. Although CNN&DCNN outperforms our models by the Precision of 78.24%, DLSTM^1^ and DLSTM^2^ achieve much higher Recall value, which means our models excel at balancing Precision and Recall. A further comparison among the LSTM-based models reveals that the multi-channel models (DLSM^1^, DLSTM^2^ and Joint AB-LSTM) give much better results in relation classification. Besides, the best performance of DLSTM^1^ can be attributed to the contribution of the dependency-based features.

**Table 4:** Performance comparison of our models with baseline methods

Models	DDI corpus								
	DrugBank			MEDLINE		Overall			
	P	R	F	P	R	F	P	R	F
DLSTM^1^	74.74	**74.57**	**74.66**	48.78	42.55	45.45	72.53	**71.49**	**72.00**
DLSTM^2^	75.29	72.64	73.95	50.67	40.43	44.97	73.29	69.54	71.37
B-LSTM	–	–	–	–	–	–	75.97	65.57	70.39
AB-LSTM	–	–	–	–	–	–	67.85	65.98	66.90
Joint AB-LSTM	–	–	–	–	–	–	73.41	69.66	71.48
CNN&DCNN	–	–	–	–	–	–	**78.24**	64.66	70.81
CNN	**77.02**	66.74	71.52	**61.43**	**45.26**	**52.12**	75.72	64.66	69.75
SCNN^2^	–	–	–	–	–	–	72.50	65.10	68.60
SCNN^1^	–	–	–	–	–	–	69.10	65.10	67.00
Kim	–	–	69.80	–	–	38.20	–	–	67.00
FBK-irst	66.70	68.60	67.60	41.90	37.90	39.80	64.60	65.60	65.10
UTurku	73.80	53.50	62.00	59.30	16.80	26.20	73.20	49.90	59.40
NIL_UCM	56.60	57.90	57.30	35.70	15.80	21.90	53.50	50.10	51.70
Models	PK DDI corpus								
	–	–	–	–	–	–	P	R	F
DLSTM^1-multi^	–	–	–	–	–	–	**89.89**	**89.89**	**89.89**
DLSTM^1-single^	–	–	–	–	–	–	87.97	87.97	87.97

Considering our models, DLSTM^1^ performs better than DLSTM^2^. It gives an indication that random word embedding is better than syntax word embedding. This may clash with the intuition that syntax word embedding should be more vital for representing a sentence’s syntactic structure than random word embedding. By statistical analysis, we can conclude that unknown words are responsible for the worse performance of DLSTM^2^. In the syntax word embedding matrix, there are 203 unknown words initialized by random values among 4279 words, leading to a break for syntax information to some extent.

The same as previous studies [6], our models perform better on DrugBank subset compared to MEDLINE subset. We observe that the sentences in MEDLINE abstracts tend to be long and complex, whereas sentences in DrugBank commonly show conciseness. In addition, one should recall that the percentage of instances from DrugBank to the training set is higher than from MEDLINE.

Moreover, for further verifying the effectiveness of DLSTM^1^, we utilize another corpus, called PK DDI corpus [43], to train our model. After preprocessing the data, 1906 instances are separated into training data and test data according to the ratio of 3:1. DLSTM^1-multi^ preserves the Linear channel, DFS channel and BFS channel, while DLSTM^1-single^ only keeps the Linear channel. As the results shown in Table 4, DLSTM^1-multi^ outperforms DLSTM^1-single^ by 1.92% of relative improvement on F-score. It gives an indication that the dependency-based channels in our model make contributions to relation classification. More narrowly, the dependency-based features extracted by going through the dependency tree using depth first search and breadth first search can better represent relation information during training our model.

### Comparison on class wise performance

As shown in Table 5, our models show the best performance for Advice, Effect and Mechanism types, whereas FBK-irst method achieves the best performance for Int type. Moreover, DLSTM^1^ outperforms all other methods by the macro-average F-score of 68.39%. Among all DDI types, Advice and Mechanism types are better identified, while Effect and Int types are more difficult to be detected by all methods. Considering the serious imbalanced training set, it is obvious that the least proportion in training data are responsible for the worst performance on Int type. This also explains the second worst performance on Effect type because of the insufficient training data.

**Table 5:** Class wise performance comparison of our models with baseline methods

Models	Advice	Effect	Mechanism	Int	MAVG
DLSTM^1^	**80.85**	68.37	**75.35**	49.00	**68.39**
DLSTM^2^	77.00	**69.47**	74.61	51.03	68.27
CNN	77.72	69.32	70.23	46.37	65.91
Kim	72.50	66.20	69.30	48.30	64.10
FBK-irst	69.20	62.80	67.90	**54.70**	64.80
UTurku	63.00	60.00	58.20	50.70	58.70
NIL_UCM	61.30	48.90	51.50	42.70	53.50

### Enhancement performance analysis

To evaluate the effectiveness of the enhancements of our model, the corresponding experiments are conducted with DLSTM^1^: an enhancement is removed or replaced each time, while -(*) denotes the removing operation and #(*) denotes the replacing operation. The effects of enhancements on performance are shown in Table 6.

**Table 6:** The effect of enhancements on performance

Enhancement removed or replaced	P	R	F	△
None	72.53	71.49	72.00	–
-DFS channel	70.22	68.21	69.20	−2.80
-BFS channel	73.52	65.23	69.13	−2.87
-DFS&BFS channels	66.57	71.69	69.04	−2.96
-Negative instance filtering	70.59	69.15	69.87	−2.13
-Train set sampling	69.51	66.67	68.06	−3.94
#Bi-LSTM outputs concatenating	70.94	66.87	68.85	−3.15

Notes. △ denotes the corresponding F-score decrease percentage when an enhancement is removed or replaced

#### DFS, BFS and DFS&BFS channels

After DFS channel enhancement and BFS Channel enhancement are removed separately, the F-scores decrease by 2.80% and 2.87%. It indicates that the features respectively extracted by going through the dependency tree using depth first search and breadth first search play similarly important roles in relation extraction. While both DFS and BFS channels are removed, the F-score decreases by 2.96%, which means handcrafted features contribute to relation classification even though such features include noise caused by natural language processing tools.

#### Negative instance filtering

removing negative instance filtering leads to the decrease of F-score by 2.13%. It shows that negative instance filtering is beneficial to our model. The negative instance filtering enhancement used in our model eliminates lots of negative instances, but almost no positive instances. 6074 out of 23,371 negative instances are removed in training set, while 1402 out of 4737 negative instances are eliminated and only 4 out of 979 positive instances are removed in test set. More than 26% negative instances are correctly filtered out, but only 0.1% positive instances are wrongly filtered out in the entire dataset.

#### Training set sampling

the training set sampling enhancement is indispensable to the relation classification as the F-score decreases by 3.94% when it is removed. Before employing under sampling and oversampling in Negative and Int instances, respectively, the ratio between Negative and Int instances is 94.0:1, while it reduces to 15.7:1 when training set sampling enhancement is set up in our model. With this enhancement, the imbalanced class distribution problem of the training set can be effectively alleviated.

#### Bi-LSTM outputs concatenating

replacing the averaging operation with concatenating operation on the output of forward LSTM layer and the output of backward LSTM layer in each channel decreases the F-score by 3.15%. It is indicated that the new simple rule of combining such outputs outperforms the rule used in the previous studies. Moreover, by averaging the outputs, the number of node in softmax layer can reduce by half, which contributes to reduce the scale of the model directly.

### Error analysis

**Fig. 4:**
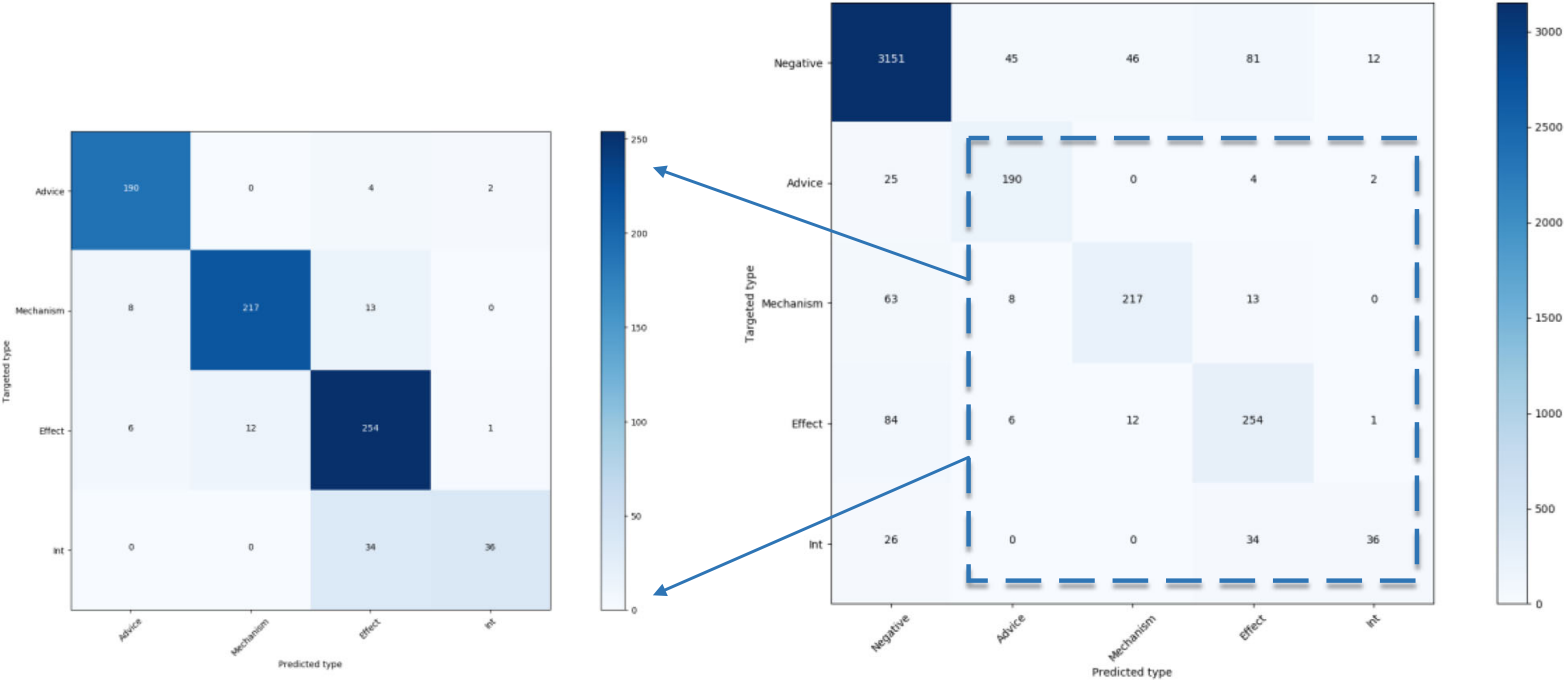
The distribution of DLSTM^1^’s predicted results for each DDI types. The vertical axis is the targeted type, while the horizontal axis is the predicted type. Point (X, Y) means the ratio, where X is predicted type and Y is targeted type. The sum of each row value equal to 1

Although our models perform better than all other methods, there still are lots of instances are wrongly classified. As shown in Fig. 4, we visualize the predicted results of DLSTM^1^ model to analyze the errors. The master diagonal region represents that the instances are predicted correctly, while the other regions reflect the distribution of error instances. As we can see from the highlighted diagonal region, DLSTM^1^ model provides a good performance on each DDI type except the Int type. Owing to the insufficient training data, the Int type is inferior in satisfying the objective function of the machine learning model. By further analysis, there is around 35.42% times that our model classifies the Effect instances into the Int instances, leading to the adverse influence on precision of the Int type.

In addition, the distribution of predicted type is relatively dispersed on the first column of Negative type. More narrowly, 198 out of 975 positive instances are wrongly detected to negative instances. It is consistent with the intuition that most of the candidate instances would be classified into negative instances due to the high proportion of negative samples in training set. Namely, the imbalanced class distribution are responsible for the low recall of DDI extraction.

**Fig. 5:**
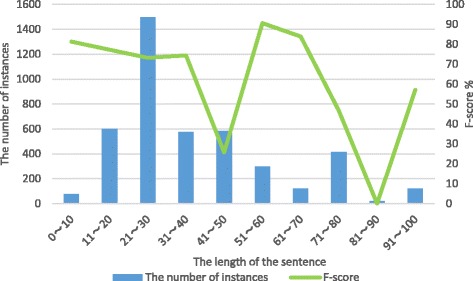
The statistic and F-score of instances with different length in test data

Furthermore, from Fig. 5, we can see that besides the imbalanced problem, the lengths of the instances adversely affect the performance of our model. Our model shows poor performance by the F-score lower than 60% when the lengths of the instances are in the range from 71 to 100, especially from 81 to 90. We observe that almost all of the instances, whose lengths are in the range from 81 to 90, are negative instances and are written in complex coordinate structure, which cannot be filtered out by negative instance filtering with limited predefined rules.

## Conclusions

In this paper, we propose a dependency-based bi-directional long short term memory network model for DDI extraction. In our model, three channels are designed to capture relation information from the distance-based features and the dependency-based features. We concatenate the outputs of these three channels, and then link it to the softmax layer to learn a DDI classifier. In addition, considering the imbalanced class distribution of the DDI corpus, we employ two enhancements to alleviate such problem, one is negative instance filtering and another is training set sampling. The experimental results have shown that our method outperforms the existing methods by new state-of-the-art performance on F-score. Moreover, our model also excels at balancing the Precision and Recall values.

For future work, we aim to adjust our model by training it on more different datasets. In addition, considering the worse performance on long and complex instances, we will try to improve our model to make it more robust.

## Abbreviations

ADR: Adverse drug reaction
Bi-LSTM: Bi-directional long short term memory network
CNN: Convolutional Neural Network
DDI: Drug-drug interaction extraction
LSTM: Long Short Term Memory Network
RNN: Recurrent Neural Network
SVM: Support Vector Machine
